# Transarterial interventions in civilian gunshot wound injury: experience from a level-1 trauma center

**DOI:** 10.1186/s42155-023-00396-5

**Published:** 2023-10-16

**Authors:** Qian Yu, Alex Lionberg, Kylie Zane, Ethan Ungchusri, Jonathan Du, Karan Nijhawan, Austin Clarey, Rakesh Navuluri, Osman Ahmed, Priya Prakash, Jeffrey Leef, Brian Funaki

**Affiliations:** 1 Department of Radiology, University of Chicago Medical Center, 5841 S Maryland Ave, Chicago, IL 60637 USA; 2 Department of Surgery, University of Chicago Medical Center, Chicago, IL 60637 USA

**Keywords:** Embolization, Gun-shot wound, Penetrating trauma, Stent-graft, Trauma

## Abstract

**Purpose:**

To assess the effectiveness of trans-arterial vascular interventions in treatment of civilian gunshot wounds (GSW).

**Materials and methods:**

A retrospective review was performed at a level-1 trauma center to include 46 consecutive adults admitted due to GSW related hemorrhage and treated with endovascular interventions from July 2018 to July 2022. Patient demographics and procedural metrics were retrieved. Primary outcomes of interest include technical success and in-hospital mortality. Factors of mortality were assessed using a logistic regression model.

**Results:**

Twenty-one patients were brought to the endovascular suite directly (endovascular group) from the trauma bay and 25 patients after treatment in the operating room (OR group). The OR group had higher hemodynamic instability (48.0% vs 19.0%, *p* = 0.040), lower hemoglobin (12.9 vs 10.1, *p* = 0.001) and platelet counts (235.2 vs 155.1, *p* = 0.003), and worse Acute Physiology and Chronic Health Evaluation (APACHE) score (4.1 vs 10.2, *p* < 0.0001) at the time of initial presentation. Technical success was achieved in all 40 cases in which targeted embolization was attempted (100%). Empiric embolization was performed in 6/46 (13.0%) patients based on computed tomographic angiogram (CTA) and operative findings. Stent-grafts were placed in 3 patients for subclavian artery injuries. Availability of pre-intervention CTA was associated with shorter fluoroscopy time (19.8 ± 12.1 vs 30.7 ± 18.6 min, *p* = 0.030). A total of 41 patients were discharged in stable condition (89.1%). Hollow organ injury was associated with mortality (*p* = 0.039).

**Conclusion:**

Endovascular embolization and stenting were effective in managing hemorrhage due to GSW in a carefully selected population. Hollow organ injury was a statistically significant predictor of mortality. Pre-intervention CTA enabled targeted, shorter and equally effective procedures.

**Supplementary Information:**

The online version contains supplementary material available at 10.1186/s42155-023-00396-5.

## Introduction

Since the inception of the COVID-19 pandemic, there has been a 15% increase in gunshot wound (GSW) incidence and 28% increase in GSW-related deaths in the United States [1]. Homicide related firearm injury resulted in a total medical cost of $175 million and $227 billion in 2020 [2]. Data from National Trauma Data Bank suggested an overall mortality of 11.5% associated with firearm injuries [3]. Endovascular interventions such as embolization and stent-grafting are well-known minimally invasive treatments in blunt trauma, and commonly employed in visceral and pelvic injuries [4–6]. However, compared to blunt trauma and non-GSW penetrating injuries, high energy bullet ballistics are complex, and multiple vessels can be damaged in a less predictable pattern [7–9]. These injuries are associated with higher morbidity and mortality and the at risk population in the United States differs significantly compared to those being treated for blunt trauma [10–13]. Currently, there is a complete absence of reported studies on endovascular series interventions in GSW, and their role remains wholly undefined with most treatment algorithms using anecdotal evidence or extrapolating from blunt trauma. The goal of the present study was to assess the effectiveness of managing civilian GSW via endovascular interventions in a carefully selected population at a single level-1 urban trauma center.

## Materials and methods

### Patient selection

An institutional review board compliant retrospective review was performed at a single-institution Level-1 trauma center. All patients were initially evaluated by a multidisciplinary trauma team in the trauma bay following Advanced Trauma Life Support (ATLS) guidelines [14]. Although somewhat nuanced based on anatomical location of GSW, physiology, and physical exam findings, in general, patients with hemodynamic instability (systolic blood pressure < 90 or mean arterial pressure < 60) refractory to blood product resuscitation, evidence of vascular injury, non-compressible hemorrhage resuscitation and/or suspicion of hollow visceral injury were transferred to the operating room (OR) for emergent exploratory laparotomy at the attending trauma surgeon’s discretion. Otherwise, patients were evaluated with a computed tomography angiogram (CTA). Patients with evidence of hemorrhage and active bleeding, arterial transection or pseudoaneurysm identified on CTA that did not meet criteria for urgent surgery (i.e. pneumoperitoneum, bowel ischemia, aortic injury, etc.) underwent endovascular assessment for treatment of vascular injury identified on CTA. In patients who underwent surgery first, persistent hemorrhage secondary to GSW at the end of surgery or in the postoperative time period for possible embolization and/or stent graft insertion. The following baseline characteristics were collected: age, sex, GSW penetrated organs, indication for intervention, hemodynamic instability upon initial presentation to the trauma bay (defined as systolic blood pressure < 90 mmHg), laboratory values at the time of intervention including hemoglobin, platelet count and international normalized ratio (INR), availability of pre-interventional imaging, angiographic findings, treated vessel, treatment material and technique. The following baseline clinical scores were calculated: Acute Physiology and Chronic Health Evaluation (APACHE) II, Injury severity score (ISS), Revised trauma score (RTS), and Trauma injury severity score (TRISS).

### Technique

All endovascular procedures were performed by a fellowship-trained attending physician with or without resident/fellow in a university hospital. Arterial access was obtained with right or left common femoral artery using a micropuncture set (Cook, Bloomington, IN, USA) as previously described [15]. Diagnostic angiograms of the local vascular territory were performed with digital subtraction angiography (DSA) based on clinical and preoperative imaging findings. In general, patients who had pre-procedure CTA underwent targeted angiography, others underwent diagnostic angiography to assess for bleeding sites. Embolization was performed when active extravasation, pseudoaneurysm, and/or arterial transection were discovered using coaxial technique with a 2.4-Fr microcatheter (Renegade® STC; Boston Scientific, Marlborough, MA; Progreat®; Terumo Corporation, Shibuya, Japan). Empiric embolization was performed on patients without angiographic abnormalities but presented with high clinical suspicion of ongoing intermittent hemorrhage based on surgical or pre-interventional CT. Embolic materials included coils (Interlock™, Boston Scientific, Natick, MA; Nester, Cook, Bloomington, IN; Ruby Coil Penumbra, Alameda, CA) and/or gelfoam (Gelfoam Sponge, Pfizer, New York, NY, USA; EmboCube, Merit Medical Systems, South Jordan, UT). Stent-grafts were considered for large vessel injury such as subclavian arteries (Viabahn, WL Gore & Assoc, Flagstaff, AZ). Examples are shown in Figs. S1, 2 and 3.

### Outcomes

Outcomes of interest include technical success (defined as cessation of contrast extravasation or obliteration of target vessel irregularity/pseudoaneurysm), clinical success (cessation of target vessel hemorrhage confirmed on post-intervention CT studies, direct visualization during surgical washout/closure, or clinical/laboratory judgment), and in-hospital mortality. Secondary outcomes include baseline characteristic and angiographic findings based on patients treated in the OR or angio-suite first, as well as fluoroscopy time (FT) and contrast volume use based on pre-interventional CT availability.

### Statistics

Data were summarized by mean/standard deviation (SD) for numerical variables and crude number/percentage for categorical variables. Baseline characteristics between patients who underwent surgery and EAS first were compared with t-tests for continuous variables and chi-square or Fisher’s exact tests for noncontinuous variables. Fluoroscopy time and contrast volume use between groups with and without pre-interventional CT were compared with t-tests. Logistic regression with Firth's penalized likelihood approach was performed to evaluate for association of baseline variables and mortality. Categorical variables were reported as counts/percentage, whereas numeric results were reported as median/range and odds ratios (ORs) with 95% confidence intervals (CI). All statistical analysis was performed with Stata 15.1 (STATA Corp., College Station, TX, USA).

## Results

**Fig. 1:**
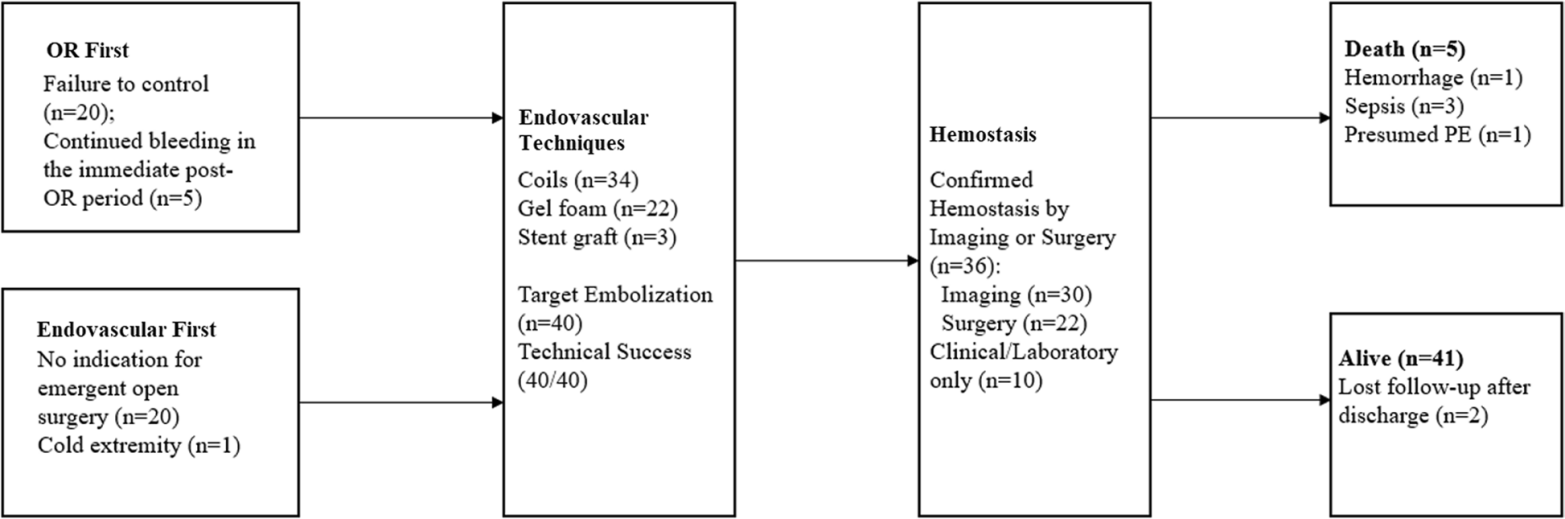
Flow-diagram of treatment and outcome

Among a total of 14,572 adult patients (≥ 18 years) presented to the trauma bay from 7/2018 to 7/2022, a total 4298 (29.5%) patients were admitted for GSW; Among 1141 GSW patients who were brought to OR directly, 25 patients were treated in EAS afterwards (OR-first group), whereas 21 patients were brought to the endovascular angio-suite (EAS) directly from trauma bay (EAS-first group, Fig. 1). A total of 46 consecutive GSW patients (Table 1) with a median age of 35 years (18–61 years) and 38/46 male (82.6%) underwent trans-arterial interventions. The OR-first group had lower hemoglobin (12.9 vs 10.1, *p* = 0.001), lower platelet count (235.2 vs 155.1, *p* = 0.003), higher proportion of hemodynamic instability (48.0% vs 19.0%, *p* = 0.040), and higher Acute Physiology and Chronic Health Evaluation II (APACHE II) score at the time of initial presentation (Table S1). Trauma injury severity score (TRISS) was also worse among patients treated in the OR first, though statistical significance was not reached (68.9% vs 56.5% *p* = 0.197). Preoperative imaging was available for all patients who were treated with an EAS-first approach (*p* < 0.001), and none had hollow organ injury (*p* < 0.001). Among 25 patients who underwent laparotomy first, 20/25 (80%) patients were brought to EAS due to failure to achieve intraoperative hemostasis, whereas 5/25 (20%) patients presented with persistent hemorrhage in the immediate post-operative period.

**Table 1:** Baseline characteristics and predictor analysis of mortality

Variables	Findings	Odds Ratio (95%CI)	*p*-value
Age	30 (18–61)	1.04 (0.96–1.13)	0.263
Sex	M: F = 38:8	0.42 (0.02–8.41)	0.569
OR first	25 (54.3%)	11.5 (0.60–222.1)	0.105
Preoperative Imaging	26/46 (56.5%)	0.22 (0.031–1.51)	0.123
Organ Injury			
Liver	27 (52.2%)	1.0 (0.18–5.67)	1.00
Spleen	6 (13.0%)	2.21 (0.28–17.25)	0.449
Kidney	7 (15.2%)	0.418 (0.02–8.41)	0.569
Hollow Organs	18 (39.1%)	23.22 (1.20–451.11)	**0.038**
Pancreas	2 (4.3%)	9.0 (0.77–105.75)	0.08
Adrenal Glands	2 (4.3%)	1.44 (0.061–34.01)	0.823
Lung/Thorax	27 (58.7%)	1.95 (0.0281.37)	0.101
Spine	6 (13.0%)	0.496 (0.024–10.11)	0.649
Shoulder/Buttock	8 (17.4%)	0.358 (0.018–7.13)	0.501
Extremities	22 (47.8%)	15.4 (0.80–297.00)	0.070
Pelvis	11 (23.9%)	2.44 (0.41–14.44)	0.324
Contrast Extravasation	35 (76.1%)	1.0 (0.14–7.21)	1.01
Treatment Material			
Coil	34 (73.9%)	4.66 (0.24–90.82)	0.310
Gel foam	22 (47.8%)	3.81 (0.54–26.67)	0.178
Stent graft	3 (6.5%)	1.00 (0.045–22.09)	1.00
Empiric Embolization	6 (13.0%)	4.74 (0.74–30.27)	0.10
Laboratory Value			
Hemoglobin	12.1 (7.0–19.0)	0.896 (0.63–1.27)	0.534
Platelet	184.5 (48–408)	1.00 (0.99–1.01)	0.772
INR	1.2 (0.9–9.3)	1.13 (0.75–1.70)	0.570
ISS	44.2 (2.4)	1.05 (0.99–1.11)	0.125
RTS	7.7 (0.5)	1.18 (1.00–1.39)	0.056
TRISS	12.4 (9.5)	0.97 (0.95–1.00)	0.066
APACHE II	7.4 (0.8)	1.16 (1.00–1.35)	0.053

*APACHE II* Acute Physiology and Chronic Health Evaluation II, *ISS* Injury severity score, *RTS* Revised trauma score, *TRISS* Trauma injury severity score

Active contrast extravasation on angiography was present in 35/46 (76.1%) patients (Fig S4). Other findings included pseudoaneurysm (*n* = 6, 13.0%), vessel contour irregularity (*n* = 1, 2.2%), dissection (*n* = 1, 2.2%), arteriovenous fistula (*n* = 1, 2.2%), and transection (*n* = 1, 2.2%). Embolization was performed in 44 patients with coils (22/44, 50.0%), gel foam (10/44, 22.7%), and both (12/44, 27.3%). Technical success was achieved in all 40 cases (100%) in which targeted treatment was attempted (targeted embolization *n* = 37, stent-graft only *n* = 2, targeted embolization and stent-graft *n* = 1). Empiric embolization was performed in 6/46 patients (13.0%, left and/or right hepatic artery = 4, intercostal arteries = 1, and internal iliac artery = 1) given high suspicion of hemorrhage in the examined region based on pre-interventional CTA or intraoperative findings. Stent-grafts were placed in three patients for subclavian artery injuries (6.5%).

**Fig. 2:**
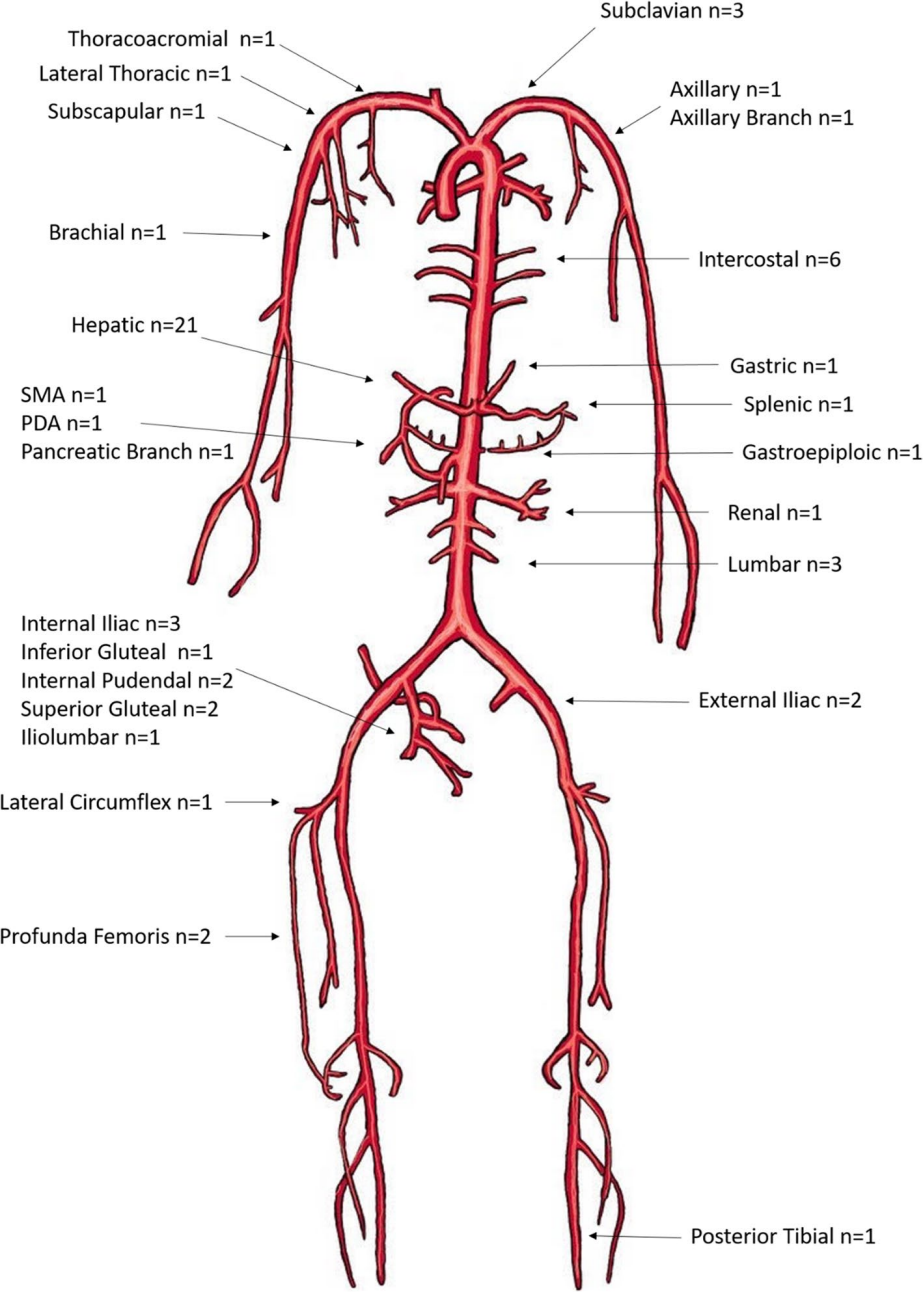
Endovascularly treated arteries. PDA: pancreaticoduodenal artery. SMA: superior mesenteric artery

The locations of treated vessels are shown in Fig. 2. Twenty-eight patients underwent treatment for a single vessel; thirteen patients were treated for two vessels; three or more vessels were treated in five patients. The vascular territories treated among the OR-first group appeared to be more diverse (Table S2) and more likely to require multiple vascular bed embolization (Table S1, *p* = 0.008). Cases with pre-intervention CT required shorter fluoroscopy time (19.8 ± 12.1 min vs 30.7 ± 18.6 min, *p* = 0.030) similarly, a lower volume of contrast material (126.6 ± 66.9 ml vs 172.8 ± 93.4 ml, *p* = 0.084) was used among patients with pre-intervention CT, though statistical significance was not reached (Table S3 ).

Post-interventional hemostasis was confirmed with cross-sectional imaging (*n* = 30) and/or during surgical washout/reoperation (*n* = 22) in 36 (78.3%) patients. In the other 10 (21.7%) patients, continued hemorrhage or suspicion for rebleeding was low based on clinical examination and hemoglobin trend. A total of 41 patients were discharged in stable condition (89.1%). Among three patients who received subclavian artery stent grafts, there was no evidence of neurovascular compromise of the upper extremity at 6, 11, and 14-month follow-up. Two patients were placed on 81 mg aspirin and 75 mg clopidogrel daily, whereas one patient only took daily aspirin.

Five patients died during index admission (10.9%). One patient with multiple GSW to the abdomen and extremities was found to have gastroduodenal artery (GDA) hemorrhage during initial laparotomy when he underwent infrarenal inferior vena cava ligation and superior mesenteric vein primary repair. This patient underwent technically successful GDA and lumbar artery embolization to stasis and then pancreatic resection and duodenal exclusion. Postoperatively, he continued to require massive transfusion, developed coagulopathy and compartment syndrome, and expired on postoperative day 2 (1/46, 2.2%). Three patients expired due to multisystemic failure related to sepsis at post-intervention day 2, 31, and 66 days, respectively. Hemostasis was confirmed at re-operation and washout in these 3 cases. Sudden death occurred in one patient at post-intervention day 40, who was stable for discharge. This patient developed tachypnea and tachycardia, followed by intubation and attempted extracorporeal membrane oxygenation insertion but developed ventricular fibrillation. While autopsy was not performed, it was suspected to be acute pulmonary embolism based on patient’s clinical presentation and history. Injury to hollow organs was associated with higher mortality (OR: 23.22 [95%CI: 1.20–451.11], *p* = 0.038). While worse revised trauma score (RTS), TRISS and APACHE scores were associated with poor survival, none of these were statistically significant (*p* = 0.056, 0.066, and 0.053, respectively). All patients were initially treated in the OR (OR: 11.5 [95%CI: 0.60–222.1], *p* = 0.105).

## Discussion

The initial management of GSW trauma is dictated by anatomical location, physiology, and physical exam findings. Patients with hemodynamic instability, unresponsive to blood product resuscitation, vascular injury, and non-compressible hemorrhage generally warrant emergent surgical intervention [16, 17] Patients that are hemodynamically stable and without indication for operative intervention at the initial evaluation typically undergo CTA imaging. Patients with vascular injuries that are difficult to access surgically can be treated with embolization or stent grafts when appropriate [18, 19]. Only a small percentage of patients in the present series received endovascular treatment (45/1162, 3.9%) compared to surgery, which underscores the importance of careful screening and illustrates that surgical intervention remains the treatment of choice in the vast majority of patients with GSW.

Nevertheless, this study demonstrates that embolization and stent grafting for GSW related hemorrhage can be highly effective with a technical success rate of 100% and in-hospital survival rate of 89.1%, which is comparable to the 88.5% survival rate based on the National Trauma Data Bank study that also included patients with minor trauma that were managed conservatively without surgery or endovascular interventions [3]. Target vessel hemostasis was achieved in all patients regardless of whether they were treated in the EAS directly from the trauma bay or as a salvage option after unsuccessful surgical hemostasis, though one patient died from exsanguination after multi-site injury despite attempted surgery and angiographically successful endovascular interventions (2.3%). Compared to patients who underwent initial exploratory laparotomy, patients that were initially managed in EAS had less severe injuries; these patients had a higher proportion of hemodynamic stability, higher hemoglobin and platelet counts at initial presentation, better APACHE scores, lower rate of hollow organ injury, and fewer vessel injuries requiring subsequent embolization.

Current society guidelines recommend a multi-disciplinary approach involving angioembolization evaluation and treatment for acute trauma [20–22]. Embolization is recommended for blunt hepatic injury with ongoing bleeding and angiographic target, as the first-line therapy for pelvic trauma over surgery, grade IV/V blunt splenic trauma, and grade III/IV renal injuries when surgical exploration is not warranted, supported by guidelines from World Society of Emergency Surgery (WSES), Society of Interventional Radiology (SIR), Eastern Association of the Surgery of Trauma (EAST), and American Urology Association [20, 23–27]. However, recommendations for endovascular treatment is mainly extrapolated from blunt trauma and stab wounds [20]; most literature on gunshot wound injuries in the last two decades is limited to case reports and small case series [28, 29]. In a 7-year analysis during Afghanistan war of 685 patients with vascular injuries, 374 diagnostic and 27 therapeutic endovascular procedures were performed, respectively, though the outcomes for these patients were not reported [30]. In the present cohort, patients who were successfully treated with embolization throughout the body including for liver, pelvis, kidney, and splenic injuries. Target hemostasis was achieved in all cases as demonstrated by follow-up cross-sectional imaging, surgical washout, and hemoglobin trending.

The present study showed effectiveness of endovascular interventions in less common regions of trauma. A total of 3/46 patients (6.5%) were treated with covered stent graft for subclavian artery transection. According to a retrospective cohort of 57 patients with penetrating subclavian artery injuries, 3% and 32% patients developed angiographic significant stenosis or occlusion occurred in short- and long-terms, respectively, which were managed either conservatively or with endovascular intervention [5]. These injuries are more difficult to repair surgically due to proximal location, and thus endovascular repair should be considered, especially in the setting of single vessel injury [31, 32]. Five patients (10.9%) from the current cohort underwent intercostal artery embolization with 100% clinical and technical success. A retrospectively cohort of 24 consecutive patients with blunt trauma that underwent intercostal artery embolization reported a primary technical success rate of 87.5% [33]. Three patients in the present study underwent lumbar artery embolization (6.5%) with 100% technical success. The present study also demonstrated that embolization in treatment of hemorrhage in muscle compartments such as the lower extremities, buttocks, and neck was effective.

Among the 5 in-hospital mortalities, bacteremia was the highest cause of deaths (3/5). Hollow organ injury was a statistically significant predictor of in-hospital mortality, likely related to the risk of developing bacteremia from bowel content spillage. Imaging and clinical signs of bowel perforation upon initial presentation warrant emergent surgery for source control. In the setting of polytrauma, surgery is commonly required to repair hollow organ damage. Meanwhile, endovascular intervention can be considered for damage control, visceral organ hemorrhage, or other areas that are less surgically accessible such as deep pelvis, retrohepatic, and posterior intercostal regions. While the present study suggested effectiveness of endovascular intervention in achieving hemostasis, long-term complications from trauma, such as sepsis, underscore the importance of source control by timely abdominal washout, identification and drainage of postoperative abscess.

From a technical perspective, availability of preoperative CTA was associated with statistically significant shorter fluoroscopy time as well as a trend of lower contrast volume use, likely due to the fact that pre-operative imaging allows for both a global assessment of injury and a targeted approach to diagnostic angiography. This approach avoids unnecessary interrogation of arteries with low risk of hemorrhage. The initial "extra" time for obtaining pre-intervention CTA appears to be justified and compensated by the ease to localize culprit vessel during catheter angiography [34]. According to a recently published retrospective study including 190 patients with abdominopelvic trauma, preoperative CT was associated with improved therapeutic embolization rate and procedure metrics such as technical success, contrast use, and post-intervention transfusion requirement regardless of patient’s hemodynamic stablility [35]. These findings advocate a CT-first approach when possible prior to catheter-directed angiography and endovascular therapy.

The present study should be interpreted with several caveats. Included patients were heterogeneous in terms of injured organ, baseline condition, and injury severity. Each trauma scenario requires distinct treatment algorithms, and operator expertise is crucial as small and large vessel endovascular interventions require different skill sets [20]. Moreover, treatment approach and outcomes of the present cohort are based on a single institutional algorithm, which may vary across different institutions, leading to selection bias. For instance, endovascular staffing and EAS may not be readily available in many institutions. In places with limited endovascular resources, such as the battlefield and most civilian hospital centers, open surgery remains to be the dominant approach even for surgically challenging anatomical locations that are otherwise easily accessible endovascularly.

## Conclusion

In summary, the present study demonstrates that endovascular interventions for civilian GSW were effective with high rates of technical success and hemostasis in appropriately chosen patients, both as initial definitive and as salvage treatment. These interventions remain secondary and complementary to surgical intervention, as the vast majority of patients with GSWs in the present series were treated surgically and increasing injury severity typically mandated operative management as the initial therapy of choice. When feasible, pre-intervention cross-sectional imaging should be obtained to guide interventional radiologists to limit both fluoroscopy and total procedure times. Hollow organ injury is a statistically significant predictor of mortality, for which clinicians should be cognizant of infection prevention and timely source control.

### Supplementary Information

**Additional file 1: Figure S1. **Left thigh gun-shot wound: A) Pre-embolization axial computed tomographic angiogram (CTA) through the level of the left thigh demonstrates an area of contrast extravasation. B) Angiogram demonstrates areas of active contrast extravasation corresponding to the areas seen on pre-embolization CTA. C: Post coil-embolization angiogram demonstrates resolution of contrast extravasation.

**Additional file 2: ** **Figure S2. **Patient with gun-shot wounds to the liver and right kidney. A) Computed tomographic angiogram (CTA) demonstrates small focal area of contrast extravasation in the inferior aspect of the right hepatic lobe. B) Hepatic arterial branches supply the inferior right hepatic lobe were evaluated and no angiographic correlate for the extravasation on CTA was found. C) Empiric gel foam embolization of the arterial branches supplying the R inferior hepatic lobe was performed. D) CTA demonstrates focal area of contrast extravasation in the kidney and surrounding hematoma. E) Angiogram of the right kidney demonstrates regions of devascularized kidney and multiple areas of contrast extravasation. F) Microcoils were used to embolize the bleeding vessels (right). 

**Additional file 3:** **Figure S3. **Right subclavian artery injury.A) Pre-interventonal computed tomography shows focal traumatic dissection flap of the right subclavian artery.B) Pre-intervention angiogram shows filling defect of the right subclavian artery compatible with dissection flap. C) Angiogram after stent-graft placement shows patency of right subclavian artery.

**Additional file 4: Figure S4. **Detailed angiographic findings and treatment approaches of included patients.

**Additional file 5:** **Supplement Table 1.** Characteristics of patients who were treated in endovascular angio-suite (EAS) or operating room (OR) first. APACHE II: Acute Physiology and Chronic Health Evaluation II. ISS: Injury severity score. RTS: Revised trauma score. TRISS: Trauma injury severity score.

**Additional file 6:** **Supplement Table 2.** Endovascularly treated vascular territories between patient groups who were treated in endovascular angio-suite (EAS) or operating room (OR) first. 

**Additional file 7:** **Supplement Table 3.** Technical variables between patients with and without pre-interventional computed tomography angiogram (CTA).

## Data Availability

Upon request.

## Abbreviations

APACHE: Acute Physiology and Chronic Health Evaluation
ATLS: Advanced Trauma Life Support
CI: Confidence interval
CT: Computed tomography
CTA: Computed tomography angiography
EAS: Endovascular angio-suite
EAST: Eastern Association of the Surgery of Trauma
FT: Fluoroscopy time
GDA: Gastroduodenal artery
GSW: Gun-shot wound
OR: Operating room
RTS: Revised trauma score
SD: Standard deviation
SIR: Society of Interventional Radiology
TRISS: Trauma injury severity score (TRISS)
WSES: World Society of Emergency Surgery
