# Supplementary figures and images for: Transarterial interventions in civilian gunshot wound injury: experience from a level-1 trauma center
Source: CVIR Endovasc. 2023 Oct 16;6:47. doi: 10.1186/s42155-023-00396-5 (PMC10579195; doi:10.1186/s42155-023-00396-5)

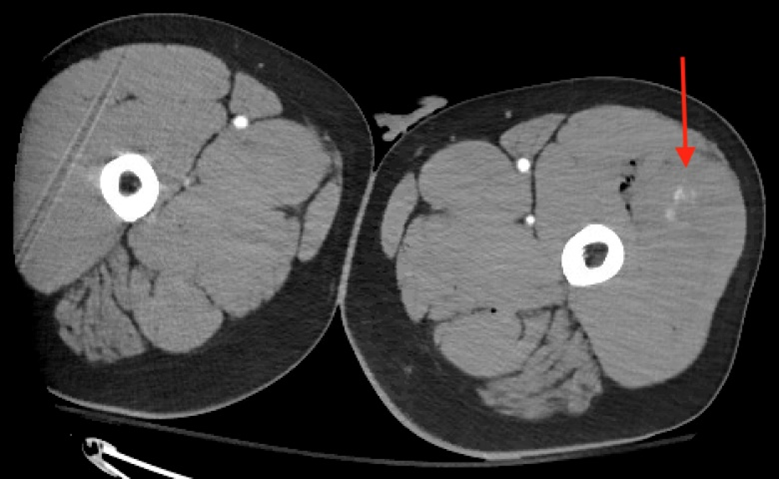

Supplement: Supplementary file 1 — Additional file 1: Figure S1. Left thigh gun-shot wound: A) Pre-embolization axial computed tomographic angiogram (CTA) through the level of the left thigh demonstrates an area of contrast extravasation. B) Angiogram demonstrates areas of active contrast extravasation corresponding to the areas seen on pre-embolization CTA. C: Post coil-embolization angiogram demonstrates resolution of contrast extravasation. [file 42155_2023_396_MOESM1_ESM.zip › S.Figure 1A400R1.tif]

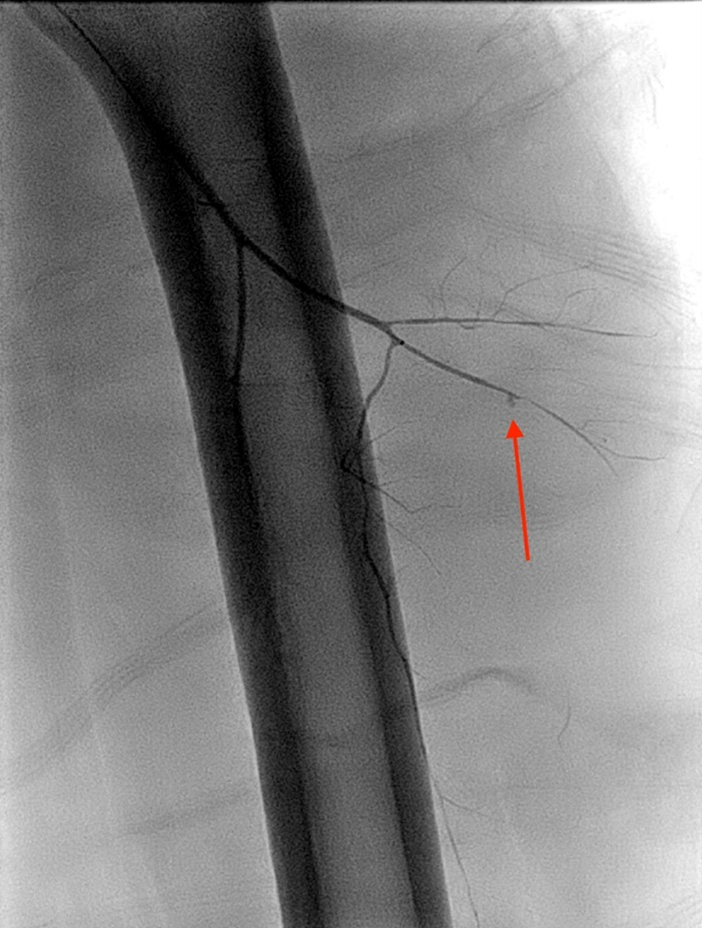

Supplement: Supplementary file 1 — Additional file 1: Figure S1. Left thigh gun-shot wound: A) Pre-embolization axial computed tomographic angiogram (CTA) through the level of the left thigh demonstrates an area of contrast extravasation. B) Angiogram demonstrates areas of active contrast extravasation corresponding to the areas seen on pre-embolization CTA. C: Post coil-embolization angiogram demonstrates resolution of contrast extravasation. [file 42155_2023_396_MOESM1_ESM.zip › S.Figure 1B400R1.tif]

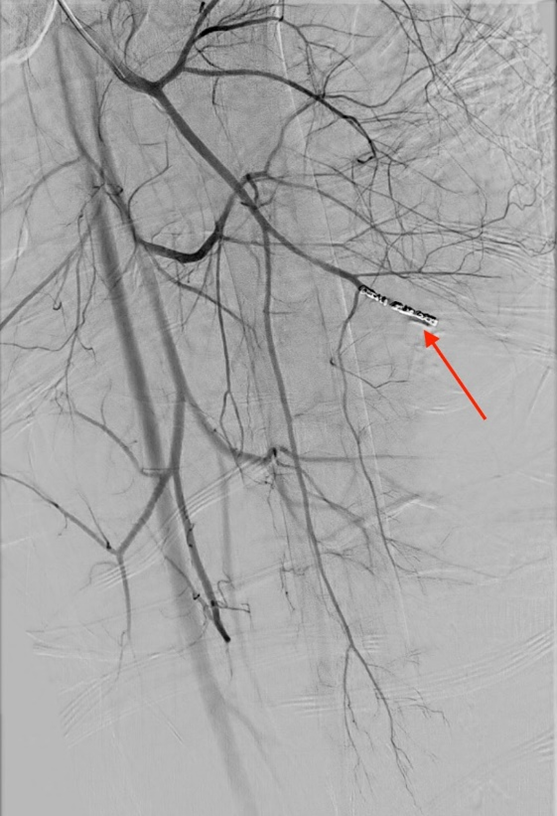

Supplement: Supplementary file 1 — Additional file 1: Figure S1. Left thigh gun-shot wound: A) Pre-embolization axial computed tomographic angiogram (CTA) through the level of the left thigh demonstrates an area of contrast extravasation. B) Angiogram demonstrates areas of active contrast extravasation corresponding to the areas seen on pre-embolization CTA. C: Post coil-embolization angiogram demonstrates resolution of contrast extravasation. [file 42155_2023_396_MOESM1_ESM.zip › S.Figure 1C400R1.tif]

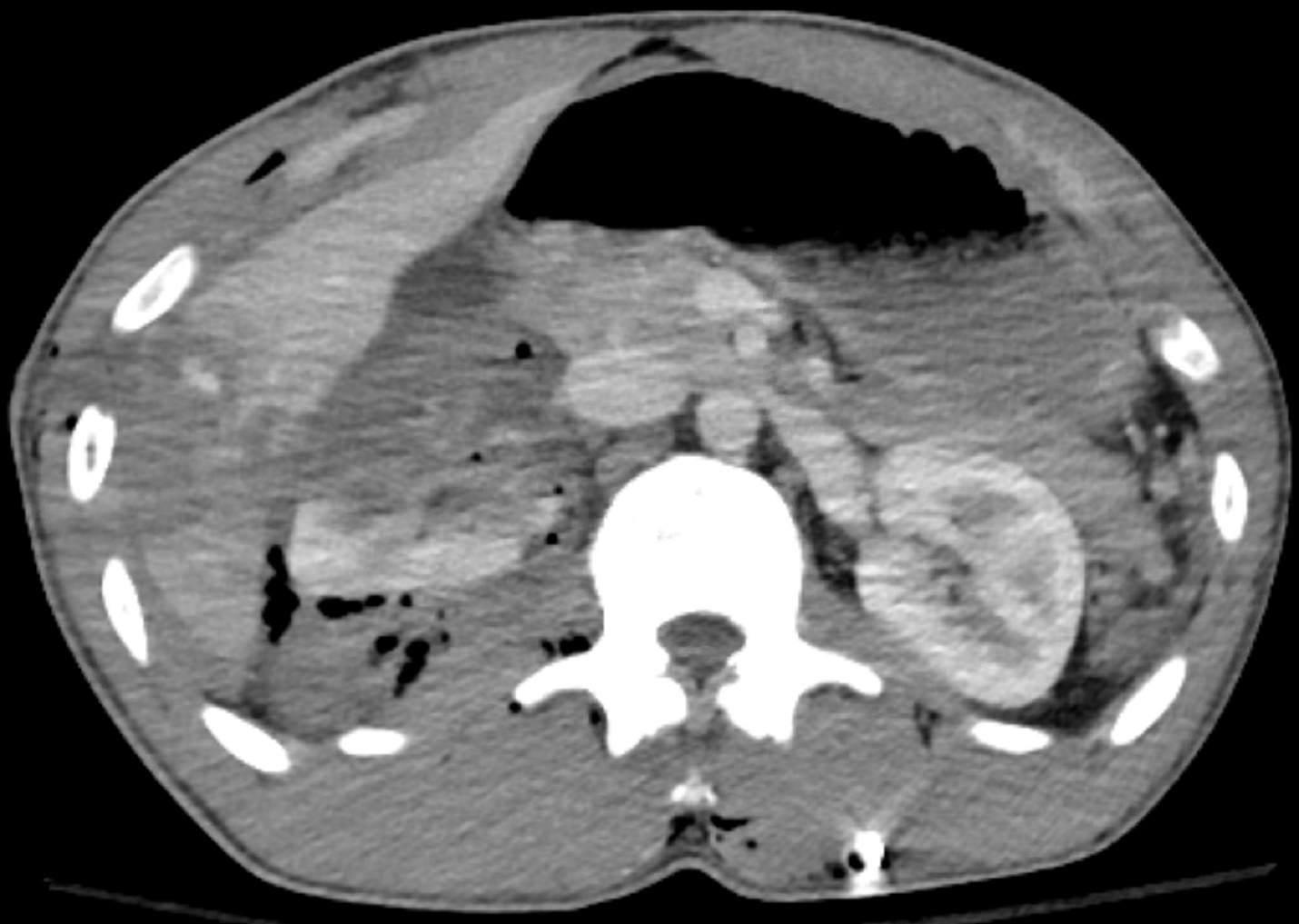

Supplement: Supplementary file 2 — Additional file 2: Figure S2. Patient with gun-shot wounds to the liver and right kidney. A) Computed tomographic angiogram (CTA) demonstrates small focal area of contrast extravasation in the inferior aspect of the right hepatic lobe. B) Hepatic arterial branches supply the inferior right hepatic lobe were evaluated and no angiographic correlate for the extravasation on CTA was found. C) Empiric gel foam embolization of the arterial branches supplying the R inferior hepatic lobe was performed. D) CTA demonstrates focal area of contrast extravasation in the kidney and surrounding hematoma. E) Angiogram of the right kidney demonstrates regions of devascularized kidney and multiple areas of contrast extravasation. F) Microcoils were used to embolize the bleeding vessels (right). [file 42155_2023_396_MOESM2_ESM.zip › S.Figure 2A400R1.tif]

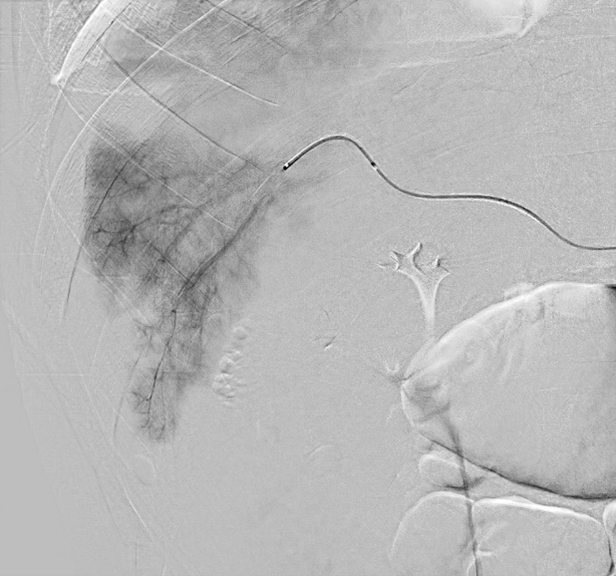

Supplement: Supplementary file 2 — Additional file 2: Figure S2. Patient with gun-shot wounds to the liver and right kidney. A) Computed tomographic angiogram (CTA) demonstrates small focal area of contrast extravasation in the inferior aspect of the right hepatic lobe. B) Hepatic arterial branches supply the inferior right hepatic lobe were evaluated and no angiographic correlate for the extravasation on CTA was found. C) Empiric gel foam embolization of the arterial branches supplying the R inferior hepatic lobe was performed. D) CTA demonstrates focal area of contrast extravasation in the kidney and surrounding hematoma. E) Angiogram of the right kidney demonstrates regions of devascularized kidney and multiple areas of contrast extravasation. F) Microcoils were used to embolize the bleeding vessels (right). [file 42155_2023_396_MOESM2_ESM.zip › S.Figure 2B400 (1)R1.tif]

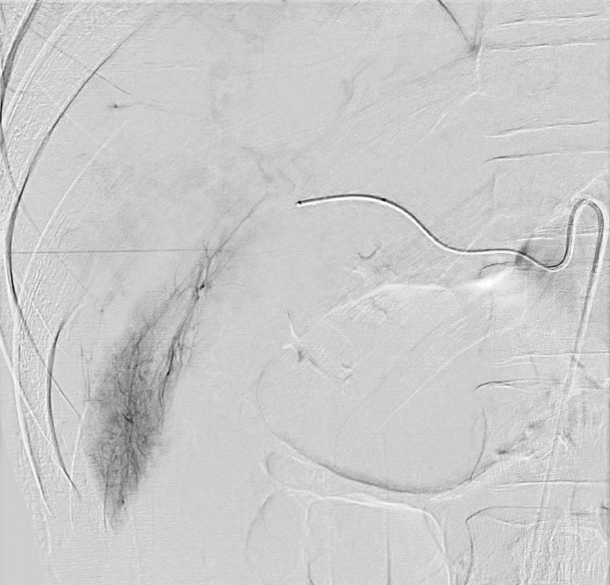

Supplement: Supplementary file 2 — Additional file 2: Figure S2. Patient with gun-shot wounds to the liver and right kidney. A) Computed tomographic angiogram (CTA) demonstrates small focal area of contrast extravasation in the inferior aspect of the right hepatic lobe. B) Hepatic arterial branches supply the inferior right hepatic lobe were evaluated and no angiographic correlate for the extravasation on CTA was found. C) Empiric gel foam embolization of the arterial branches supplying the R inferior hepatic lobe was performed. D) CTA demonstrates focal area of contrast extravasation in the kidney and surrounding hematoma. E) Angiogram of the right kidney demonstrates regions of devascularized kidney and multiple areas of contrast extravasation. F) Microcoils were used to embolize the bleeding vessels (right). [file 42155_2023_396_MOESM2_ESM.zip › S.Figure 2C400R1.tif]

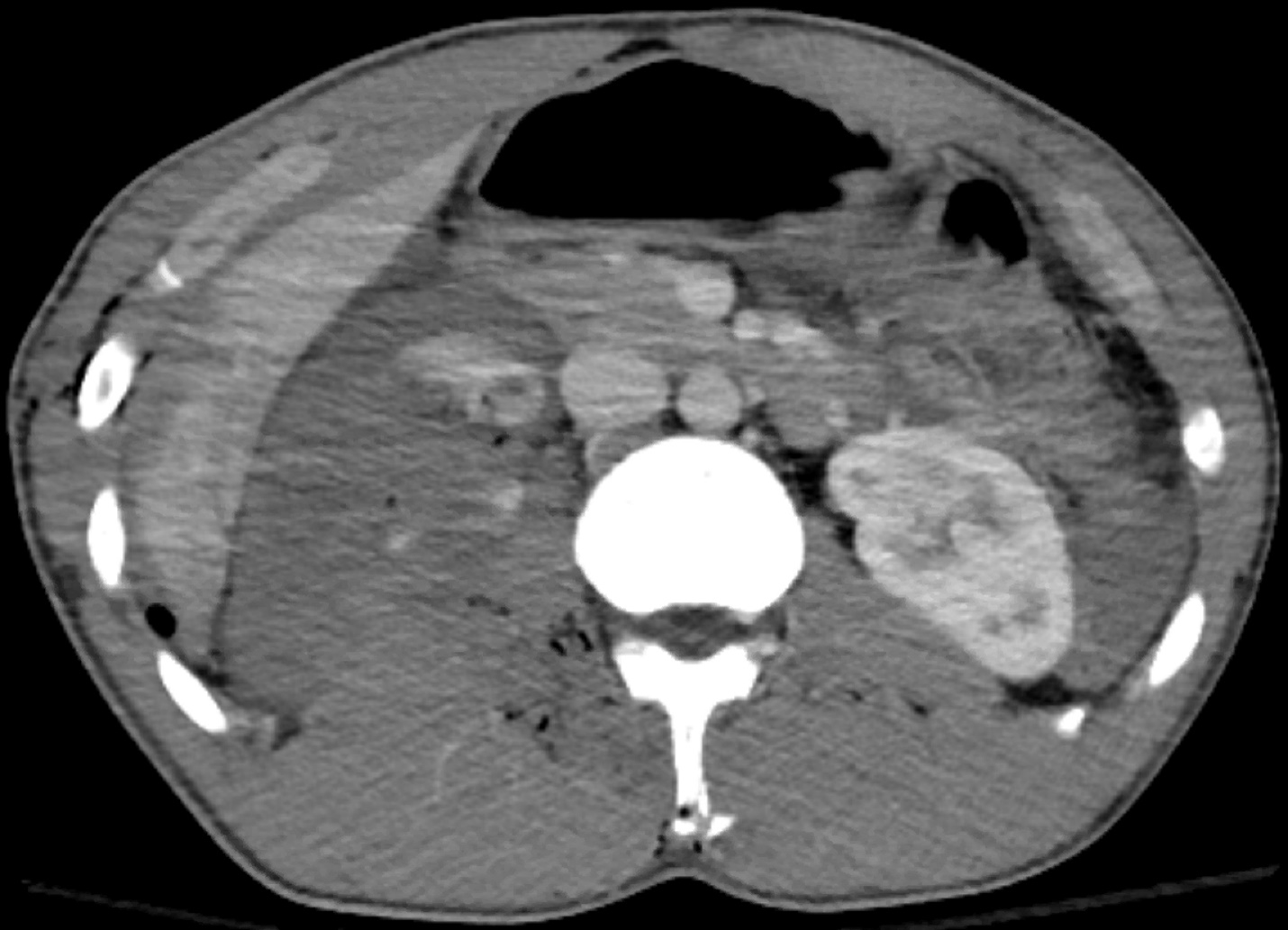

Supplement: Supplementary file 2 — Additional file 2: Figure S2. Patient with gun-shot wounds to the liver and right kidney. A) Computed tomographic angiogram (CTA) demonstrates small focal area of contrast extravasation in the inferior aspect of the right hepatic lobe. B) Hepatic arterial branches supply the inferior right hepatic lobe were evaluated and no angiographic correlate for the extravasation on CTA was found. C) Empiric gel foam embolization of the arterial branches supplying the R inferior hepatic lobe was performed. D) CTA demonstrates focal area of contrast extravasation in the kidney and surrounding hematoma. E) Angiogram of the right kidney demonstrates regions of devascularized kidney and multiple areas of contrast extravasation. F) Microcoils were used to embolize the bleeding vessels (right). [file 42155_2023_396_MOESM2_ESM.zip › S.Figure 2D400R1.tif]

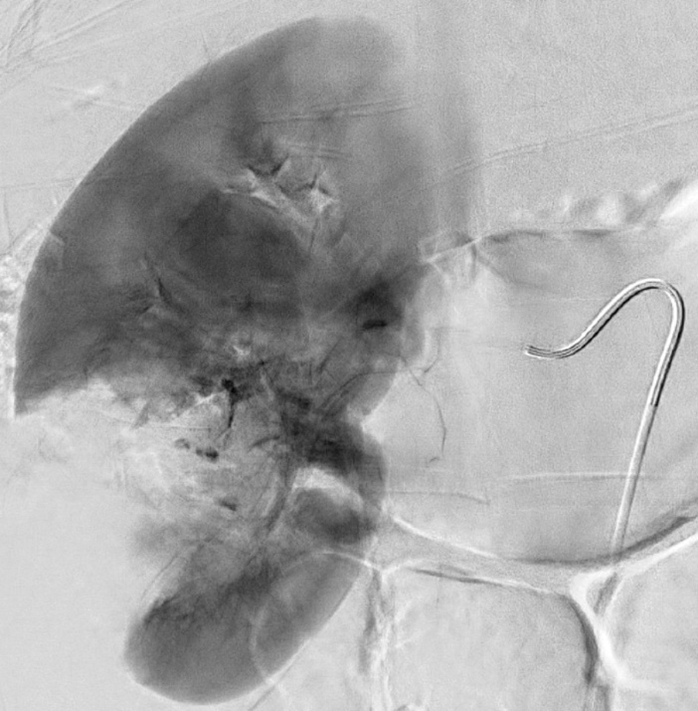

Supplement: Supplementary file 2 — Additional file 2: Figure S2. Patient with gun-shot wounds to the liver and right kidney. A) Computed tomographic angiogram (CTA) demonstrates small focal area of contrast extravasation in the inferior aspect of the right hepatic lobe. B) Hepatic arterial branches supply the inferior right hepatic lobe were evaluated and no angiographic correlate for the extravasation on CTA was found. C) Empiric gel foam embolization of the arterial branches supplying the R inferior hepatic lobe was performed. D) CTA demonstrates focal area of contrast extravasation in the kidney and surrounding hematoma. E) Angiogram of the right kidney demonstrates regions of devascularized kidney and multiple areas of contrast extravasation. F) Microcoils were used to embolize the bleeding vessels (right). [file 42155_2023_396_MOESM2_ESM.zip › S.Figure 2E400R1.tif]

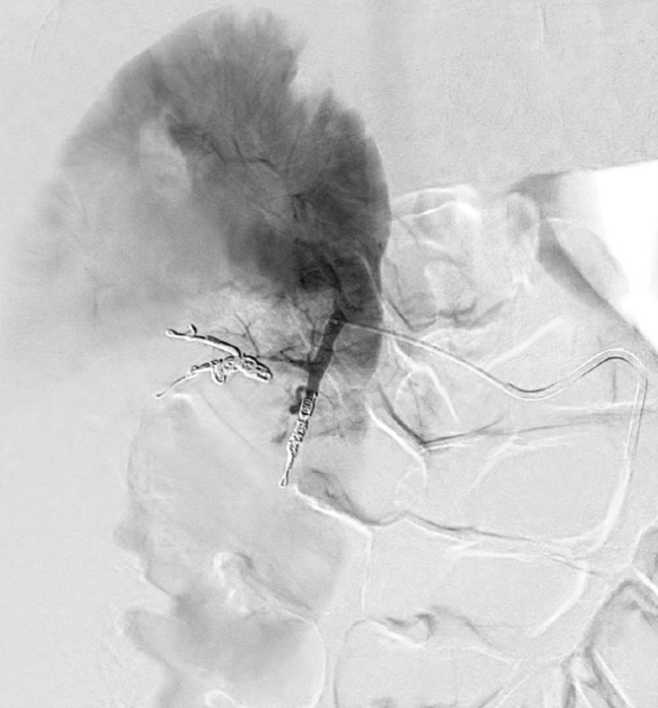

Supplement: Supplementary file 2 — Additional file 2: Figure S2. Patient with gun-shot wounds to the liver and right kidney. A) Computed tomographic angiogram (CTA) demonstrates small focal area of contrast extravasation in the inferior aspect of the right hepatic lobe. B) Hepatic arterial branches supply the inferior right hepatic lobe were evaluated and no angiographic correlate for the extravasation on CTA was found. C) Empiric gel foam embolization of the arterial branches supplying the R inferior hepatic lobe was performed. D) CTA demonstrates focal area of contrast extravasation in the kidney and surrounding hematoma. E) Angiogram of the right kidney demonstrates regions of devascularized kidney and multiple areas of contrast extravasation. F) Microcoils were used to embolize the bleeding vessels (right). [file 42155_2023_396_MOESM2_ESM.zip › S.Figure 2F400R1.tif]

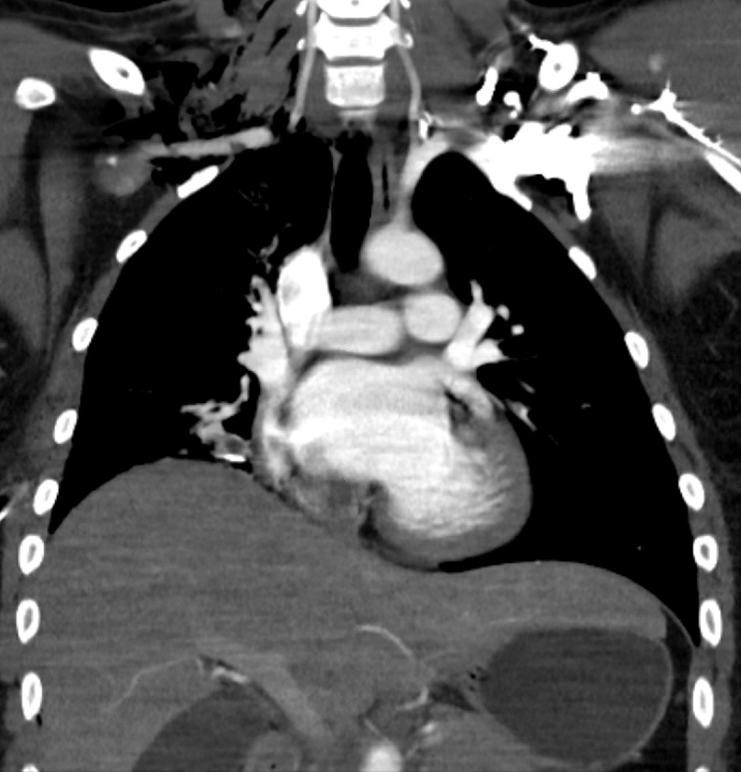

Supplement: Supplementary file 3 — Additional file 3: Figure S3. Right subclavian artery injury.A) Pre-interventonal computed tomography shows focal traumatic dissection flap of the right subclavian artery.B) Pre-intervention angiogram shows filling defect of the right subclavian artery compatible with dissection flap. C) Angiogram after stent-graft placement shows patency of right subclavian artery. [file 42155_2023_396_MOESM3_ESM.zip › S.Figure 3A400R1.tif]

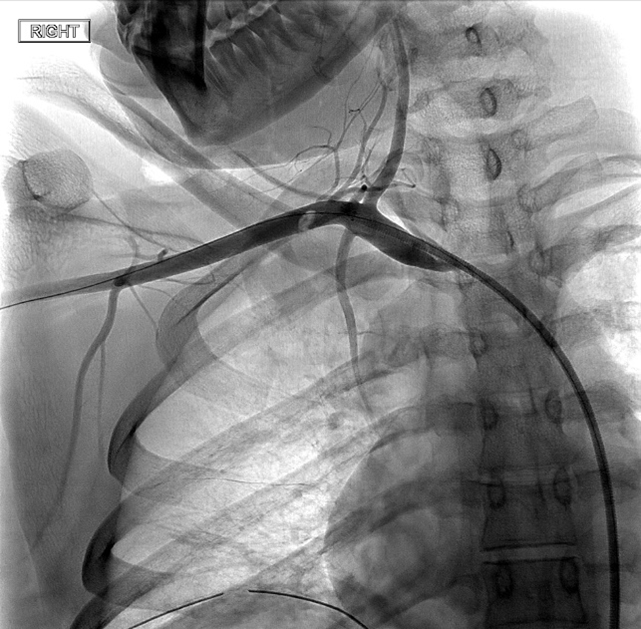

Supplement: Supplementary file 3 — Additional file 3: Figure S3. Right subclavian artery injury.A) Pre-interventonal computed tomography shows focal traumatic dissection flap of the right subclavian artery.B) Pre-intervention angiogram shows filling defect of the right subclavian artery compatible with dissection flap. C) Angiogram after stent-graft placement shows patency of right subclavian artery. [file 42155_2023_396_MOESM3_ESM.zip › S.Figure 3B400R1.tif]

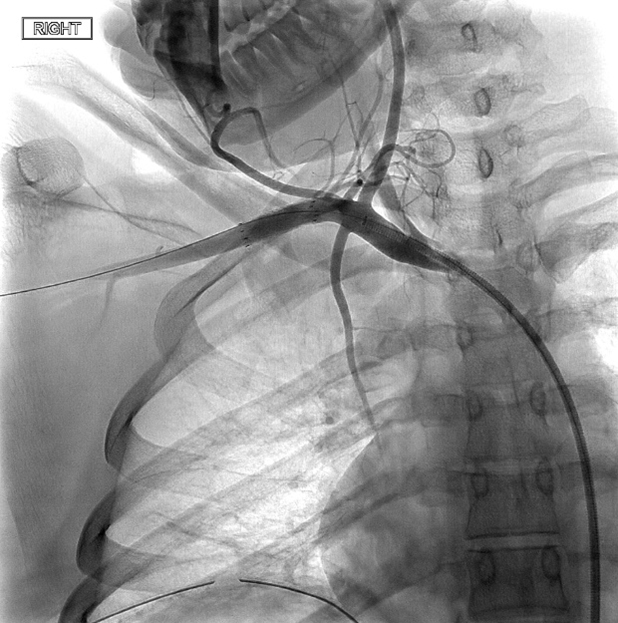

Supplement: Supplementary file 3 — Additional file 3: Figure S3. Right subclavian artery injury.A) Pre-interventonal computed tomography shows focal traumatic dissection flap of the right subclavian artery.B) Pre-intervention angiogram shows filling defect of the right subclavian artery compatible with dissection flap. C) Angiogram after stent-graft placement shows patency of right subclavian artery. [file 42155_2023_396_MOESM3_ESM.zip › S.Figure 3C400R1.tif]

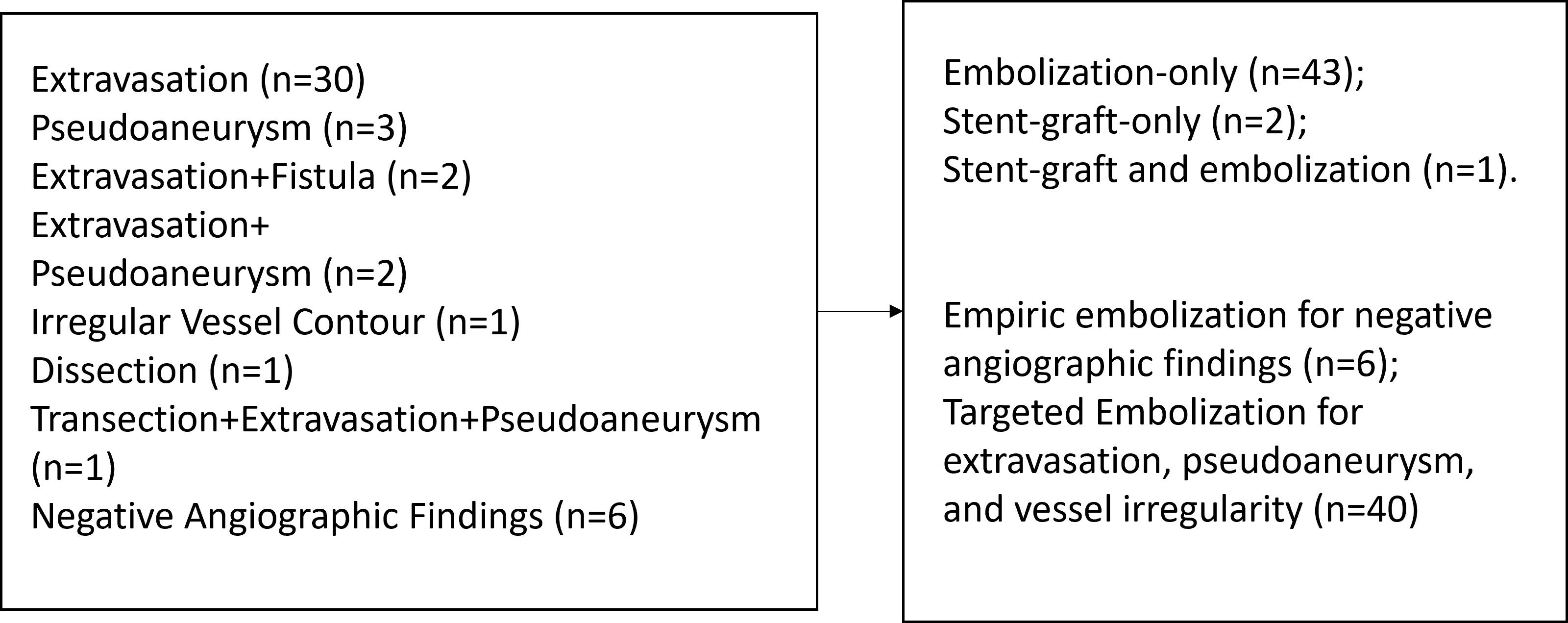

Supplement: Supplementary file 4 — Additional file 4: Figure S4. Detailed angiographic findings and treatment approaches of included patients. [file 42155_2023_396_MOESM4_ESM.tif]
