# Supplementary material for: Transarterial interventions in civilian gunshot wound injury: experience from a level-1 trauma center
Source: CVIR Endovasc. 2023 Oct 16;6:47. doi: 10.1186/s42155-023-00396-5 (PMC10579195; doi:10.1186/s42155-023-00396-5)
Supplement: Supplementary file 5 — Additional file 5: Supplement Table 1. Characteristics of patients who were treated in endovascular angio-suite (EAS) or operating room (OR) first. APACHE II: Acute Physiology and Chronic Health Evaluation II. ISS: Injury severity score. RTS: Revised trauma score. TRISS: Trauma injury severity score. [file 42155_2023_396_MOESM5_ESM.docx]

| Variables | EAS first (n=21) | OR first (n=25) | p-value |
| --- | --- | --- | --- |
| Age | 31.2 (11.8) | 32.0 (8.8) | 0.835 |
| Sex (M: F) | 17:4 | 22: 3 | 0.102 |
| Hemodynamic Instability | 4/21 (19.0%) | 12/25 (48.0%) | **0.040** |
| Preoperative Imaging | 21/21 (100%) | 5/25 (20.0%) | **<0.001** |
| Organ Injury  Liver  Spleen  Kidney  Hollow Organs  Lung/Thorax  Shoulder/Buttock  Extremities  Pelvis | 10  0  3  0  1  5  8  3 | 12  3  3  10  6  1  8  4 | 0.083  0.064  0.677  **<0.001**  0.112  0.367  0.364  0.418 |
| Laboratory Value:  Hemoglobin  Platelet  INR | 12.9 (11.8)  235.2 (72.5)  1.2 (0.6) | 10.1 (9.0)  155.1 (78.6)  1.3 (0.2) | **0.001**  **0.003**  0.710 |
| ISS  RTS  TRISS  APACHE II | 43.19 (3.53)  7.71 (0.122)  68.90 (6.89)  4.10 (0.61) | 45.04 (3.38)  7.64 (0.86)  56.50 (6.46)  10.24 (1.11) | 0.7083  0.9378  0.1969  **<0.0001** |
| Fluoroscopy Time | 19.6 (13.4) | 24.3 (14.9) | 0.267 |
| Multi-vessel embolization (3 or more treated vascular territories) | 0 | 5/25 (20.0%) | **0.008** |

**Supplement Table 1**: Characteristics of patients who were treated in endovascular angio-suite (EAS) or operating room (OR) first. APACHE II: Acute Physiology and Chronic Health Evaluation II. ISS: Injury severity score. RTS: Revised trauma score. TRISS: Trauma injury severity score.
