# Supplementary material for: Transarterial interventions in civilian gunshot wound injury: experience from a level-1 trauma center
Source: CVIR Endovasc. 2023 Oct 16;6:47. doi: 10.1186/s42155-023-00396-5 (PMC10579195; doi:10.1186/s42155-023-00396-5)
Supplement: Supplementary file 6 — Additional file 6: Supplement Table 2. Endovascularly treated vascular territories between patient groups who were treated in endovascular angio-suite (EAS) or operating room (OR) first. [file 42155_2023_396_MOESM6_ESM.docx]

|  | EAS first | OR first | P value |
| --- | --- | --- | --- |
| Upper Thoracic/Shoulder  Axillary  Thoracoacromial  Subclavian  Lateral thoracic  Subscapsular | 5 (22.7%)  2 (9.1%)  1 (4.5%)  2 (9.1%)  0  0 | 3 (7.7%)  0  0  1 (2.6%)  1 (2.6%)  1 (2.6%) | 0.124 |
| Intercostal | 1 (4.5%) | 5 (12.8%) | 0.404 |
| Arm/Brachial | 1 (4.5%) | 0 | 0.361 |
| Legs  Tibial  Profundus Femoris | 2 (9.1%)  1 (4.5%)  1 (4.5%) | 1 (2.6%)  0  1 (2.6%) | 0.293 |
| Pelvis  Internal iliac artery  Internal Pudendal  Circumflex  External iliac artery  Superior gluteal  Iliolumbar  Inferior gluteal | 3 (13.6%)  1 (4.5%)  1 (4.5%)  0  1 (4.5%)  0  0  0 | 9 (23.1%)  2 (5.1%)  1 (2.6%)  1 (2.6%)  1 (2.6%)  2 (5.1%)  1 (2.6%)  1 (2.6%) | 0.509 |
| Abdominal  Hepatic  Renal  Gastric  SMA  Spleen  Gastroepiploic  Pancreatic  PDA  Lumbar | 10 (45.5%)  9 (40.9%)  1 (4.5%)  0  0  0  0  0  0  0 | 21 (53.8%)  12 (30.8%)  0  1 (2.6%)  1 (2.6%)  1 (2.6%)  1 (2.6%)  1 (2.6%)  1 (2.6%)  3 (7.7%) | 0.662 |
| Total Vascular Territories | 22 | 39 |  |

**Supplement Table 2**: Endovascularly treated vascular territories between patient groups who were treated in endovascular angio-suite (EAS) or operating room (OR) first.
