# Supplementary material for: Transarterial interventions in civilian gunshot wound injury: experience from a level-1 trauma center
Source: CVIR Endovasc. 2023 Oct 16;6:47. doi: 10.1186/s42155-023-00396-5 (PMC10579195; doi:10.1186/s42155-023-00396-5)
Supplement: Supplementary file 7 — Additional file 7: Supplement Table 3. Technical variables between patients with and without pre-interventional computed tomography angiogram (CTA). [file 42155_2023_396_MOESM7_ESM.docx]

| Variables | CTA (n=36) | No CTA (n=10) | p-value |
| --- | --- | --- | --- |
| Fluoroscopy Time (s) | 19.8 (12.1) | 30.7 (18.6) | 0.030 |
| Contrast Use (ml) | 126.6 (66.9) | 172.8 (93.4) | 0.084 |

**Supplement Table 3**: Technical variables between patients with and without pre-interventional computed tomography angiogram (CTA).
